# Effects of Repetitive Peripheral Sensory Stimulation in the Subacute and Chronic Phases After Stroke: Study Protocol for a Pilot Randomized Trial

**DOI:** 10.3389/fneur.2022.779128

**Published:** 2022-02-16

**Authors:** Jéssica Borges Kroth, Benjamim Handfas, Glaucia Rodrigues, Francisco Zepeda, Marco Aurélio Oliveira, Danny J. J. Wang, Raymundo Machado de Azevedo Neto, Gisele Sampaio Silva, Edson Amaro, Isaac Olubunmi Sorinola, Adriana Bastos Conforto

**Affiliations:** ^1^Hospital Israelita Albert Einstein, São Paulo, Brazil; ^2^Biological Engineering Department, Massachusetts Institute of Technology, Boston, MA, United States; ^3^Department of Neurology, Keck School of Medicine, University of Southern California, Los Angeles, CA, United States; ^4^Department of Physiotherapy, King's College London, London, United Kingdom

**Keywords:** sensory stimulation, stroke, rehabilitation, upper limb, nerve stimulation

## Abstract

**Background:**

Repetitive peripheral nerve sensory stimulation (RPSS) is a potential add-on intervention to motor training for rehabilitation of upper limb paresis after stroke. Benefits of RPSS were reported in subjects in the chronic phase after stroke, but there is limited information about the effects of this intervention within the 1st weeks or months. The primary goal of this study is to compare, in a head-to-head proof-of-principle study, the impact of a single session of suprasensory vs. subsensory RPSS on the upper limb motor performance and learning in subjects at different phases after stroke subacute and chronic phases and mild upper limb motor impairments after stroke. In addition, we examine the effects of RPSS on brain perfusion, functional imaging activation, and γ-aminobutyric acid (GABA) levels. Subjects with mild upper limb motor impairments will be tested with MRI and clinical assessment either at an early (7 days to 3 months post-stroke) or at a chronic (>6 months) stage after stroke.

**Methods:**

In this multicenter, randomized, parallel-group, proof-of-principle clinical trial with blinded assessment of outcomes, we compare the effects of one session of suprasensory or subsensory RPSS in patients with ischemic or hemorrhagic stroke and upper limb paresis. Clinical assessment and MRI will be performed only once in each subject (either at an early or at a chronic stage). The primary outcome is the change in performance in the Jebsen–Taylor test. Secondary outcomes: hand strength, cerebral blood flow assessed with arterial spin labeling, changes in the blood oxygenation level-dependent (BOLD) effect in ipsilesional and contralesional primary motor cortex (M1) on the left and the right hemispheres assessed with functional MRI (fMRI) during a finger-tapping task performed with the paretic hand, and changes in GABA levels in ipsilesional and contralesional M1 evaluated with spectroscopy. The changes in outcomes will be compared in four groups: suprasensory, early; subsensory, early; suprasensory, chronic; and subsensory, chronic.

**Discussion:**

The results of this study are relevant to inform future clinical trials to tailor RPSS to patients more likely to benefit from this intervention.

**Trial Registration:**

NCT03956407.

## Introduction

Upper limb paresis is very common after stroke, a leading cause of disability worldwide (1, 2). Interventions based on intensive, repetitive tasks training can improve motor performance (3). Over the past two decades, neuromodulation interventions, such as repetitive transcranial magnetic stimulation (TMS) (4), transcranial direct current stimulation (5), and repetitive peripheral sensory stimulation (RPSS) (6), have been investigated as potential add-on therapies to boost the effects of training in subjects with stroke.

Repetitive peripheral sensory stimulation, in particular, is a straightforward intervention designed to enhance somatosensory input. In RPSS, trains of electric pulses are delivered to peripheral nerves by surface electrodes with parameters of stimulation aimed to predominantly activate proprioceptive and large cutaneous sensory fibers (6). The intensity of stimulation is set to elicit paresthesia but minimal or absent motor responses. RPSS targets a brain sensorimotor network known to be physiologically relevant for motor performance and learning (7). Sensory information is relayed from peripheral nerves to the spinal cord, then, *via* the dorsal medial lemniscus in the brainstem, reaches the thalamus and finally, the sensorimotor cortex. Increased excitability, activity, or use-dependent plasticity of the motor cortex have been reported in healthy subjects (8–10) or subjects with stroke (11–13) after 2 h of RPSS. The connections between neurons that represent the motor area of the stimulated body segment may be strengthened, leading to enhancement of motor performance or learning (9, 14–16). Disinhibition of silent synapses by alterations in glutamatergic receptors or reduction of GABAergic inhibition, as well-activation of silent synapses and modulation of neuronal interactions (17) may mediate these effects. The presence of functional and structural connections between areas that are responsible for motor control (16) and its relationship with somatosensory afferents underlies the rationale for the use of RPSS in individuals with motor impairment, such as stroke (18).

In subjects in the chronic phase after stroke, the effect size of RPSS to improve upper limb motor function is similar to or greater than the effect sizes of other neuromodulation interventions (6). On the other hand, information about the effects of RPSS in the subacute phase after stroke is limited. While all studies in chronic subjects with mild-to-moderate upper limb motor impairments showed benefits of this intervention, only two addressed effects in the subacute stage: one reported an increase in thumb strength after one session of RPSS compared to stimulation to the level of perception (19), whereas another described more significant improvement after *subsensory* RPSS compared to *suprasensory* RPSS (20). In patients in the chronic phase after stroke, suprasensory stimulation led to greater improvements than subsensory stimulation, and the latter was used as a control intervention in patients in the chronic phase after stroke (21, 22). Therefore, the finding of greater effects of subsensory stimulation, compared to suprasensory stimulation in patients in the subacute phase (20), was surprising and raised the hypothesis that optimal intensities/doses of stimulation may be different early, compared to at a later stage after stroke. Until now, patients in the subacute and chronic stages have not been included in a single study, in order to perform head-to-head comparisons between the effects of subsensory and suprasensory stimulation at these two phases after stroke.

Neuronal excitability, activity, or connectivity undergo changes over time after stroke. For instance, motor and sensory representations near the lesion may be remapped after cortical infarcts (23). Connectivity between primary sensorimotor and secondary motor areas changes over several weeks (24). Intracortical inhibition assessed with TMS may decrease in the contralateral motor cortex early after a cerebellar stroke and increase in the chronic phase, more than 6 months later (25).

Since excitability changes over time after lesion onset, and effects of neuromodulation interventions are known to be state-dependent (19), it is expected that results of these interventions vary at different stages after stroke. It is possible that, early after lesion onset, the variability in activity or excitability during the dynamic process of spontaneous recovery contributes to a less consistent effect of neuromodulation interventions. A systematic review and meta-analysis indicated that the potential benefits of transcranial direct current stimulation might be greater in patients in the chronic than in the subacute stage after stroke (26, 27). Furthermore, constraint-induced movement therapy (CIMT), a rehabilitation intervention that increases afferent input and provides training to decrease learned non-use of the upper limb, seems to be more beneficial in the chronic than in the subacute phase after stroke considering that the EXCITE trial (28) showed significant benefits of CIMT in patients treated between 3 and 9 months post-stroke, whereas the VECTORS (29) study showed no differences in effects of this intervention, compared to usual care, on average within the first 10 days after stroke.

Data from TMS studies suggest that RPSS enhances cortical excitability in animals (30), healthy subjects (31, 32), and patients in the chronic phase after stroke affecting the corticospinal tract (24). RPSS, compared to no stimulation (sham), leads to an increase in signal intensity and in the number of voxels activated in the primary motor cortex (M1) during thumb movements, in a paradigm of functional MRI (fMRI) based on the blood oxygen level-dependent (BOLD) effect. Perfusion in M1 assessed with arterial spin labeling at rest in healthy subjects increases after RPSS (7). This finding may be explained by increased blood flow driven by enhanced neuronal activity in M1. Whether RPSS can increase BOLD activation or global/regional cerebral blood flow (CBF) in subjects with stroke at different stages remains to be determined.

Likewise, the effects of RPSS on synaptic activity or concentration of neurotransmitters are unknown. γ-Aminobutyric acid in the sensorimotor cortex has been implicated in the processing of afferent input. For instance, in healthy subjects, GABA concentrations evaluated with magnetic resonance spectroscopy predict changes in perceptual outcome after afferent, tactile stimulation of fingertips (33). In rats, GABAergic inhibition modulates responsiveness to afferent stimulation of the forepaw (34). The GABA concentration or activity in M1 may influence responsiveness to RPSS. Furthermore, RPSS may also increase or decrease GABA levels in the cortex. Until now, these hypotheses have not yet been tested.

The primary goal of this study is to compare the effects of a single session of RPSS on motor performance and motor learning in subjects with stroke in the subacute (EARLYgroup) and chronic (CHRONICgroup) phases with mild upper limb motor impairments. We hypothesize that enhancement of motor performance and motor learning by RPSS will be significantly greater in subjects in the CHRONICgroup than in subjects in the EARLYgroup, when exposed to the same experimental paradigm. In addition, we compare the effects of a single session of RPSS on cerebral perfusion, changes in the BOLD effect, and on GABA levels changes in ipsilesional and contralesional M1. Our hypothesis is that changes in these outcomes will be significantly greater after RPSS in subjects in the chronic, than in the early stage.

## Methods and Analysis

### Study Design

**Figure 1:**
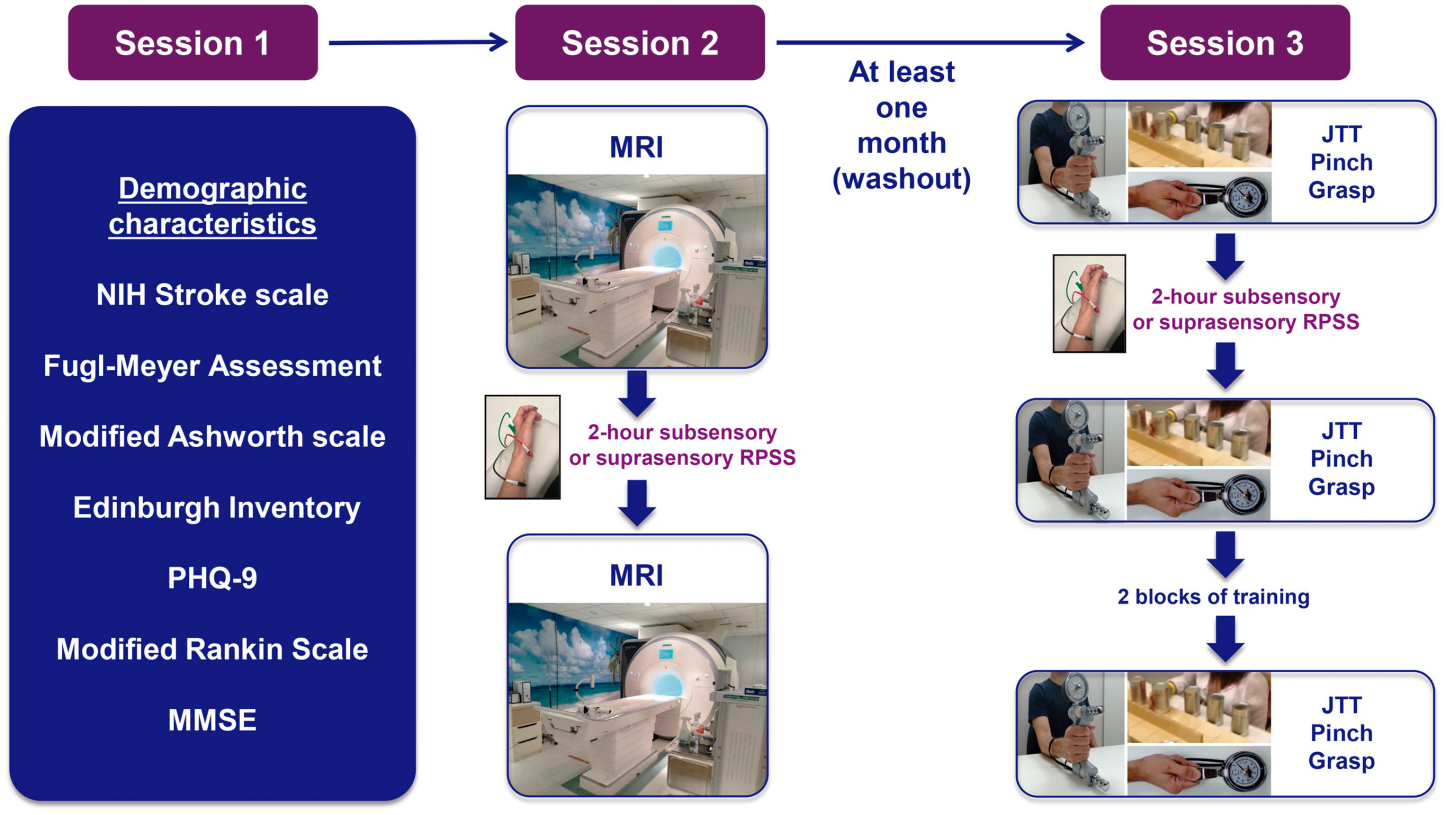
Experimental paradigm. NIH, national institutes of health; PHQ-9, patient health questionnaire-9; MMSE, mini-mental state exam; MRI, magnetic resonance imaging; RPSS, repetitive peripheral nerve sensory stimulation; JTT, jebsen–taylor test.

This is a multicenter, randomized, parallel-group, proof-of-principle clinical trial with blinded assessment of outcomes. Figure 1 summarizes the study protocol.

#### Location and Setting

This ongoing study is conducted in three hospitals in Brazil: Hospital Israelita Albert Einstein (HIAE, coordinating center), Hospital das Clínicas da Faculdade de Medicina da Universidade de São Paulo (HCFMUSP) both in São Paulo, and Hospital São Rafael (HSR) in Salvador. Initially, the study would involve two centers (HIAE and HSR). HCFMUSP was included as an additional center due to difficulties in patient recruitment. The coordinating center (HIAE) was responsible for the design of the protocol, submission of the project to the HIAE Ethics Committee and to the funding agency (Fundação de Amparo à Pesquisa do Estado de São Paulo, FAPESP), creation of the research forms, and management of the project. It is also responsible for the submission of amendments, for providing and assessing the training of researchers involved in the protocol, and for communication with all centers. Weekly meetings were performed between researchers from the three centers.

The first patient was included in December 2019. The study was interrupted in March 2020 due to the COVID-19 pandemic. Recruitment was restarted in São Paulo in May 2021 and in Salvador in July 2021.

The protocol is reported according to the Standard Protocol Items: Recommendations for Intervention Trials (SPIRIT) statement (Supplementary Materials).

### Participants

The inclusion and exclusion criteria are shown in Table 1. Subjects able to perform at least four of the tasks of the Jebsen–Taylor test (JTT) are included. The JTT scores the time (in seconds) required to perform activities often used in daily living: copying a sentence; turning over cards; picking up small common objects such as coins, bottle-cap, and paper clips; simulated feeding using a spoon; stacking checkers; and moving large light and heavy objects (32, 35).

**Table 1:** Inclusion and exclusion criteria.

**Inclusion criteria**	**Exclusion criteria**
• Age ≥ 18 years • Ischemic or hemorrhagic stroke confirmed by computed tomography or magnetic resonance imaging, between 7 days and 3 months before enrollment (subacute phase), and at least 6 months (chronic phase) • Ability to perform at least 4 of 7 tasks performed in daily life that is part of the Jebsen–Taylor test; • Upper limb paresis contralateral to the lesion	• Inability to provide consent • Anesthesia of the paretic hand • Severe spasticity at the paretic elbow, fist or fingers, defined with a score larger than 3 on the Modified Ashworth Scale • Shoulder pain or join deformity in the paretic limb • Lesions affecting cerebellum, or cerebellar pathways in the brainstem • Uncontrolled psychiatric disease • Neurological diseases such as Parkinson disease or chronic uncontrolled chronic disease such as cancer or cardiac insufficiency • Aphasia or severe cognitive deficit

### Recruitment

Subjects are recruited in the community through advertisements on websites or social media, and from admissions to HSR, HIAE, and HCFMUSP. In the three hospitals, preliminary eligibility is assessed according to information from medical records. Information about the protocol is also sent to physicians or therapists from these three institutions and from other hospitals or clinics, to be disseminated to patients who may be interested in participating in the study.

### Subjects' Characteristics

The following characteristics are evaluated at baseline in the first experimental session (Figure 1): age, gender, ethnicity, years of education, time from stroke, type of stroke (ischemic/hemorrhagic), medications, lesion side/location, etiology of ischemic strokes according to the Causative Classification System (36) and scores in the following scales: the Modified Rankin Scale, National Institutes of Health Stroke Scale (NIHSS) (37), Modified Ashworth Scale (MAS—elbow, wrist, and finger joints) (38), Oldfield Inventory (prior to stroke) (39), Fugl-Meyer Assessment of Sensorimotor recovery (upper limb—FMA) (40), Mini-Mental State Examination (37), Patient Health Questionnaire-9 (PHQ-9) (41), and Edinburgh Handedness Inventory (39). Assessments are performed by researchers (therapists or medical students). Prior to the onset of patient recruitment, all researchers had to be certified in performance of the NIHSS (https://www.nihstrokescale.org/). After availability of a certification of the Portuguese version of the MRS (https://docs.google.com/forms/d/e/1FAIpQLSfJIzi6G9SH8VaPxqe7txcK55ApfFRVvReWV37FRaARG6PbYQ/viewform), all researchers involved in the MRS assessment were certified. A video with demonstrations of how to administer and rate each item of the FMA was produced, watched, and discussed in meetings with all researchers, prior to realistic simulations in which the researchers played roles of assessors and “patients.” Data collection only started in each center after the local principal investigator considered that characteristics of the patients and outcomes had been consistently evaluated in the realistic simulations. After the beginning of the COVID-19 pandemic, data were not collected for several months. All researchers involved in the assessment of characteristics of patients or outcomes were trained again before data collection was restarted.

An experienced radiologist defines lesion locations by the evaluation of MRI (FLAIR, T2, and T1-weighted images—see “Imaging protocol”) as: right/left; frontal/temporal/occipital/parietal/insular; corticosubcortical, cortical, or subcortical; involving or not the precentral gyrus, postcentral gyrus, centrum semiovale, corpus callosum, posterior limb of the internal capsule, thalamus, basal ganglia, mesencephalon, pons, or medulla.

At HIAE, the presence of motor-evoked potentials (MEPs) to TMS is assessed by the principal investigator, who has been trained and performed the procedure for the past 20 years, in the absence of contraindications (42). A safety screening questionnaire is filled before the procedure (43). TMS is delivered to the affected hemisphere through a figure-of-eight-shaped coil (MC B-70, outer diameter 100 mm, max dB/dt31 kT/s near the coil surface) held by an investigator, connected to a biphasic MagPro X100 (Alpine Biomed). Electromyography (EMG) activity is recorded at rest from the surface electrodes placed over the *abductor pollicis brevis* muscle in the paretic hand. EMG responses are amplified (×1,000), filtered (2 Hz−2 kHz) and sampled at 5 kHz with a computerized data acquisition system built with the LabVIEW graphical programming language (44). The intervals between TMS pulses are randomized between 5 and 7 s. If no MEPs are registered at 100% of the stimulator's output, they are considered “absent.”

### Randomization, Allocation Concealment, and Blinding

Patients are randomized to one of four groups: suprasensory or subsensory in the EARLYgroup, suprasensory or subsensory in the CHRONICgroup. The randomization sequence was created by a statistician in an Excel spreadsheet in 12 blocks of 4 patients at HIAE or HCFMUSP and in 6 blocks of 4 patients at HSR, weighted 1:1 toward the suprasensory treatment group. Randomization was stratified by phase after stroke (subacute or chronic), gender, age (18–50, 51–79, ≥80 years), side of upper limb paresis and baseline upper limb FMA scores (≤47/66 or >47/66). Randomization tables are kept in locked cabinets accessed only by the principal investigator and the researcher responsible for RPSS in each center, and password-protected files.

Researchers responsible for the evaluation of outcomes are blinded to treatment allocation. All researchers received training before the onset of data collection. Videos with instructions for administration of the JTT were produced, and realistic simulations were performed. Patients are not informed about the type of stimulation they receive (suprasensory or subsensory) and are naive to the experimental hypothesis. They are instructed not to share their perceptions about the experimental sessions with other participants and researchers.

### Interventions

Suprasensory or subsensory RPSS was initially administered in the second experimental session (Figure 1) at HSR and at HIAE, and also in the third experimental session at HIAE. After approval of an amendment in August 2021, RPSS started to be administered in the first visit, after the assessment of the patient characteristics. This protocol corresponds to version 5 of the project and includes all the amendments approved until August 2021.

Subjects are comfortably seated during RPSS. Trains of electrical pulses (1 ms of duration for each) at a frequency of 10 Hz are delivered at 1 Hz (500 ms on and 500 ms off) to the median nerve on the wrist by one pair of surface electrodes (Kendall; cathode, proximal) connected to a customized stimulator (Alfamedic Ltda, São Paulo, Brazil; maximum output, 130 V). The median nerve is stimulated between the tendons of the flexor carpi radialis and palmaris longus with the cathode positioned 2–3 cm proximal to the wrist. Three measurements of the minimum stimulation intensity required to elicit paresthesia (sensory threshold) are performed.

For suprasensory stimulation, intensities are set at the highest intensity able to induce paresthesia without overt muscle contraction or pain and adjusted if required (21, 39, 45). In subsensory, the intensity of stimulation is below sensory threshold, not sufficient to elicit paresthesia (20).

Every 5 min, participants are asked about the sensations elicited by stimulation in order to avoid fluctuations in wakefulness and in attention to the stimulated body part (46). The intensity of stimulation is increased if paresthesia becomes weaker, and lowered if they become uncomfortable or during the 2-h period. Usually, it is necessary to adjust the intensity of stimulation due to changes in the intensity of paresthesia during the 2-h period. There may be changes in skin impedance and subjects in the RPSS group may report increase or decrease in paresthesia. The intensity of stimulation is then adjusted to elicit strong paresthesia in the absence of pain or movement. In the subsensory group, the initial intensity defined so that no sensations are elicited may lead to paresthesia during the 2-h period. When this occurs, the intensity of stimulation is lowered below the sensory threshold. During RPSS, subjects watch videos of their choice.

A single session of RPSS has been shown to improve motor performance in the absence of training (19–22, 47) and to enhance effects of training (11, 16, 18, 22). In session 3 (Figure 1), patients are initially familiarized with the JTT by performing each task once in two blocks of practice. Then, baseline assessments are performed. After suprasensory RPSS or subsensory for 2 h, the JTT scores and strength are reassessed. Then, the subjects undergo two blocks of training of tasks of the JTT under the supervision of a rehabilitation professional blinded to the type of stimulation received (22). During motor training, subjects are instructed to perform the JTT tasks that they can complete as fast as possible. Two blocks of training are administered. In each block, each task is performed once. JTT performance is reassessed after training.

Adverse events are monitored during and immediately after the stimulation. Studies included in a systematic review did not report any serious adverse events (6). Two studies described contact dermatitis on the skin under the electrode. If hyperemia is identified after stimulation, this will be reported as an adverse event. If it resolves spontaneously in 30 min, no further action is taken. If there is itching or persistent hyperemia, a topical corticoid cream may be used. In all three centers, the visits and experiments take place inside hospitals with medical resources. A specific insurance policy for clinical trials was taken for financial protection of the sponsor and researchers.

The visual analog scale (VAS) assessed before and after stimulation and after training, to grade sleepiness, fatigue, and anxiety (42).

### Outcome Measures

#### Primary Outcome: Change in Motor Performance After Stimulation

The primary outcome is change in the JTT performance after stimulation, compared to baseline (Figure 1): (JTT pre-stimulation – JTT post-stimulation/JTT pre stimulation) × 100. The task of copying a sentence is not performed. Previous studies showed that RPSS can enhance JTT performance and motor learning in subjects in the chronic phase after stroke (20, 29).

#### Secondary Outcomes

Change in motor performance after training JTT is also assessed after two blocks of training (Figure 1). Changes in JTT after stimulation + training are calculated as: [(JTT post-stimulation + training – JTT pre-stimulation)/(JTT pre-stimulation)] × 100.

##### Hand Strength

Lateral pinch and grasp strength are measured with a dynamometer (Saehan grip) according to a protocol with established validity (44). In Session 3 (Figure 1), patients are initially familiarized by performing lateral pinch and grisp strength once, using the dynamometer. Then, for outcome assessments, five trials of pinch and handgrip are performed before and after stimulation (Figure 1). During each trial, the patient is instructed to perform the movement for 5 s. The average of five trials is calculated. Improvement in pinch strength after RPSS was previously described in separate studies that included patients in the subacute (8) and chronic (39) post-stroke phases (44).

##### Imaging Outcomes

Before and after RPSS, GABA+ (GABA plus coedited macromolecules) levels, CBF assessed with arterial spin labeling, and task-related BOLD fMRI activation are assessed at HIAE. MRI is performed before and after suprasensory RPSS or subsensory on a 3T Magnetom PRISMA scanner (Siemens Medical Solutions, Erlangen, Germany) using a 64-channel 1H receive-only head coil, without contraindications.

A standard questionnaire is used to assess potential exclusion criteria for MRI (Supplementary Materials). The following sequences are acquired: MEGA-PRESS (48), BOLD fMRI (TR: 2,000 ms, TE: 25 ms, matrix 84 × 84, FOV 210 mm, number of slices: 42, slice thickness: 2.5 mm, voxel: 2.5 mm) and perfusion (3D GRASE Pseudo-Continuous Arterial Spin Labeling, pCASL; TR: 4,300 ms, TE: 36.76 ms, bandwidth: 2,604 Hz/Px, label time 1,500 ms, post-labeling delay: 2,000 ms). In addition, before RPSS a high resolution T1 volumetric (voxel size: 0.5 × 0.5 × 1 mm^3^, matrix size: 256 × 256, field of view: 256 × 256 mm^2^, TR: 2,500 ms, TE: 3.47 ms, TI: 1,100 ms, flip angle: 7 degrees) and after, DWI (voxel size: 2 mm, isotropic; TR:10,200 ms; TE:103 ms) and GRE (voxel size: 0.9 mm × 0.9 mm × 5 mm, matrix size: 256 × 192, field of view: 240 mm, TR: 250 ms, TE:15 ms) are acquired.

The GABA spectroscopy data are collected with MEGA-PRESS (30 × 30 × 30 mm^3^ voxel, TR 2,000 ms, TE 68 ms; water suppression band set to 4.7 ppm, and an editing band alternated between 1.9 ppm and 7.5 ppm in even and odd acquisitions; 192 averages-−96 “edit off” and 96 “edit on”) and a water reference scan using the MEGA-PRESS sequence without MEGA water suppression. Data are collected first from the right and then from the left cerebral hemispheres. Therefore, the ipsilesional and contralesional hemispheres are assessed. In each hemisphere, the region of interest is centered on the “hand knob” in the primary motor cortex (49). Patients are instructed to keep their eyes closed during imaging acquisition.

During fMRI scans, six blocks of finger tapping followed by rest are performed. Before being scanned, subjects practice a self-paced rhythmic finger tapping task consisting of 6 epochs of 30 s of finger tapping alternated with epochs of 30 s rest. Patients are instructed to keep their eyes open. Performance of finger tapping is not one of the inclusion criteria but because patients must be able to perform the JTT in order to be included, only patients with mild upper limb impairments participate in the study. All patients are encouraged to try to perform the task as accurately as possible. It is possible that some patients, despite mild motor impairments, are not able to accurately perform finger tapping. In order to check task performance, finger tapping during fMRI is videotaped. Two researchers review all the videos. Data from patients unable to perform finger tapping are excluded from the analysis.

Sleepiness can potentially influence task performance and GABA levels. Subjects are instructed not to drink alcohol 48 h before the tests, sleep the night before, drink the usual amount of coffee, and do not perform vigorous physical activity on the day of the test. Before MRI (Figure 1), the Epworth Sleepiness Scale is evaluated (50). VAS scores for sleepiness, fatigue, and anxiety (51), blood pressure, and heart rate are assessed before and after MRI.

### Imaging Data Analysis

#### GABA Spectroscopy

Data processing for the GABA quantification is carried out using Gannet v. 3.1.5, an open-source software coded with MatLab (52). Ratios of GABA levels between the affected and unaffected hemispheres, before and after suprasensory or subsensory RPSS, will be compared (53) in the EARLY and the CHRONIC groups. The pipeline for analysis is shown in Supplementary Materials.

##### pCASL Data

Post-processing of pCASL data is performed offline using a Java-based software package called CereFlow (Translational MRI, LLC, Los Angeles, CA, USA). First, label and control paired ASL images are corrected for motion and physiological noise using principal component analysis (54). Subsequently, pairwise subtraction between label and control images is performed, averaging to generate the mean difference dataset. CBF is then calculated using the standard pCASL model recommended by ASL white paper (55). The computed CBF maps are then normalized into a canonical space of the Montreal Neurological Institute template. Once normalized, an additional template of cerebral vascular territories is applied to extract the average CBF values in the following regions: leptomeningeal and perforating anterior cerebral artery, leptomeningeal and perforating middle cerebral artery, posterior cerebral artery, anterior choroidal artery, and posterior communicating artery on both (56). Furthermore, the Alberta Stroke Program Early CT Score system is applied to extract average CBF and arterial transit time (ATT) values in M1—M6 perfusion territories, the caudate nucleus, the lentiform nucleus, and the internal capsule (57, 58).

##### FMRI Pre-processing

The FMRI data processing is performed using FMRI Expert Analysis Tool (FEAT) Version 6.00 in FSL^1^ High-resolution registration to the MNI152 standard space image is carried out using FLIRT (59, 60). To properly register patient's brain images into standard space, we use spatial normalization using cost function masking (61), first registering functional images into patients' T1 images, and then to MNI 152 standard space using a lesion mask to down-weight affected tissue during the registration process. Lesion masks will be hand-drawn by a researcher who received extensive training in how to perform this procedure. The following pre-statistics processing is applied: motion correction using MCFLIRT (60), slice-timing correction using Fourier-space time-series phase-shifting, non-brain tissue removal using BET (62), spatial smoothing using a Gaussian kernel of FWHM 5 mm, grand-mean intensity normalization of the entire 4D dataset by a single multiplicative factor, and high-pass temporal filtering (Gaussian-weighted least-squares straight line fitting, with sigma = 120 s). Time-series statistical analysis is carried out using FILM with local autocorrelation correction (62).

#### Sample Size

Considering the SD of the data from Conforto et al. (22), it was estimated that to assess the primary outcome with a power of 80%, 68 patients should be included in the protocol. Considering an expected 5% dropout rate, the sample size was increased to 72. Half of the patients will have <6 months post-stroke and half, at least 6 months. Forty-eight patients will be included at HIAE or HCFMUSP and 24 at HSR.

##### Data Monitoring

The assessments of behavioral outcomes are videotaped. Since May 2021, all the data have been entered in electronic case report forms built in the REDCap platform (redcap.einstein.br). The data registered in paper forms before the onset of the COVID-19 pandemic were also entered in the platform. The forms detail all the procedures and definitions used in the protocol. The principal investigator oversees the conduct of the study, has meetings with the researchers at least once a week, and reviews the data at least once a month. All data are stored in secure servers in the research centers, with password and computer access restricted to researchers involved in the project.

Quality of imaging data is checked after each MRI by the author BH. Quality of ASL data is also checked by the author DJJW before analysis.

All the procedures in the protocol must follow Good Clinical Practice guidelines. Every 6 months, an independent Research Integrity Committee audits all the documents from the protocol, including consent forms, case report forms, standard operating procedures, and reports submitted to the Ethics Committee.

The local principal investigators from the three centers are given access to the data sets available on the password-protected REDCap platform. To ensure confidentiality, data shared with all the project team members are blinded from any identifying participant information.

### Statistical Analysis

Mean (±SD) or median (ranges) is presented according to the data distribution. Normality is tested with the Shapiro–Wilk test. Changes in JTT scores, strength, GABA levels, and CBF are compared between the four groups with general linear models or generalized estimating equation models with factors INTERVENTION (suprasensory/subsensory) and GROUP (subacute/chronic) according to the distribution of the data. The *p*-values below 0.05 are considered statistically significant.

For perfusion analyses, ratios between CBF in the affected/unaffected hemisphere and ratios between CBF in each region of interest in the affected/unaffected hemisphere are assessed. Due to the exploratory nature of these analyses, corrections for multiple comparisons are not performed.

For fMRI, a statistical analysis is implemented using a general linear model approach. Activity in ipsilesional and contralesional M1 during finger tapping is modeled by 30 s boxcar convolved with a double gamma function regressor. We also include a regressor with the first temporal derivative of the main regressor and nuisance regressors derived from the extended motion parameters. Two first-level analyses per participant are run, one pre-stimulation and one post-stimulation. Second level of analysis is carried to estimate the within-subject contrast between pre- and post-stimulation using a fixed-effects model, by forcing the random effects to zero in FLAME (FMRIB's Local Analysis of Mixed Effects) (63–65). At the third level of analysis, we estimate contrasts for each group of participants using one-sample *t*-tests to verify if there were effects of stimulation and independent *t*-tests to see changes between groups using a mixed effects model in FLAME (FMRIB's Local Analysis of Mixed Effects) stage 1 (63–65). Z (Gaussianized T/F) statistic images are thresholded using clusters determined by *Z* > 3.1 and a corrected cluster significance threshold of *p* = 0.05 (66). If there is a significant difference in change in motor performance (JTT or strength) between suprasensory and subsensory groups in either the chronic or subacute stage after stroke, bivariate analyses are performed (regressions or chi-square tests) to assess the relationship between each of these variables at baseline, and the magnitude of the improvement in behavior: baseline JTT or strength, age, sex, the intensity of RPSS (relative to sensory threshold), extent of BOLD activation (number of voxels and signal intensity) in M1, CBF (global and regional), or GABA concentration in M1 at baseline. Multiple regression is performed to assess the effects of variables if *p*-values are below 0.1 in bivariate analyses 0.05 (66).

## Discussion

Effective telerehabilitation options are deeply needed due to the constraints that the COVID-19 pandemic have imposed on face-to-face treatment. RPSS is a strong candidate as an add-on therapy to the upper limb motor training in this context because it provides neuromodulation utilizing a portable device. Operation of the device is straightforward. If future clinical trials can confirm the benefits of this intervention to subjects with stroke, and if RPSS can be safely administered by patients, relatives, or caregivers, in line with the results from Dos Santos-Fontes et al. (67), a novel tool for home-based neurorehabilitation may emerge for clinical practice. The results of this proof-of-principle study in which a single session of stimulation is delivered are relevant to plan clinical trials in which several sessions of stimulation are administered, as performed in other studies that assessed effects of RPSS in outpatient facilities (20, 68–71).

The first studies about interventions' effects on motor rehabilitation in stroke were published more than two decades ago. Yet, the evidence of clinical benefit for these interventions is scarce, and doubts have been shed about their future as therapeutic strategies (72). Major reasons for the gap between studies performed in neuromodulation laboratories and evidence-based rehabilitation practice are insufficient comprehension of mechanisms underlying different interventions, and the inclusion of patients with heterogeneous characteristics in proof-of-principle studies and clinical trials. Stroke is a heterogeneous condition and a “one-size-fits-all” approach is unlikely to significantly impact motor rehabilitation (73–75).

Examples of success of therapeutic approaches that target particular types of patients can be found in studies that led to novel reperfusion treatments in the hyperacute phase after stroke. For instance, after several trials that failed to prove benefit of mechanical thrombectomy (76), studies that included novel devices and narrowed eligibility criteria according to specific neurobiological hypotheses according to clinical and imaging characteristics showed that, for patients more likely to benefit, mechanical thrombectomy is a game-changing strategy (77).

This study aims to compare the effects of a single session of RPSS on learning and motor performance of patients after stroke in two different stages—chronic and subacute. The results of this study will be critical to help close one of the gaps about effects of RPSS on the performance and motor learning in subjects with stroke. They will be relevant to inform future clinical trials based on mechanisms to tailor RPSS to patients more likely to benefit from this intervention. This goal is achieved by comparing behavioral effects according to time after stroke (primary outcome) and by providing preliminary evidence of mechanisms underlying these effects (secondary outcomes). If there is a relation between time after stroke, GABA levels, BOLD activation or CBF measured with ASL, and responsiveness to RPSS as an add-on treatment to motor training, these factors should be considered to select patients for clinical trials.

One limitation of this study is that only patients with mild-to-moderate upper limb impairments are included. This strategy was adopted because this is a proof-of-principle study and eligibility criteria were narrowed to test a specific hypothesis. More work will be necessary to test whether the effects of RPSS differ according to the time after stroke, in patients with moderate-to-severe upper limb motor impairments.

Another potential limitation of this study is the choice of the control stimulation. There is no consensus about the best control or sham condition for RPSS. According to a standard definition, “sham is an arm type in which a group of participants receives a procedure or device that appears to be the same as the actual procedure or device being studied but does not contain active processes or components”^2^ We opted for subsensory stimulation—the stimulator is on, but subjects do not have paresthesia. Other possible controls are no stimulation (with or without placement of electrodes on the skin) or leg stimulation (11, 12, 14). A disadvantage of no stimulation is that subjects may conclude that they are not receiving active treatment, to the lack of paresthesia at any point during the experiment. A limitation of subsensory stimulation is that, in the 1st weeks after stroke (44), this intervention may have similar effects to stimulation above sensory threshold in the chronic phase. A caveat of stimulation of lower limb nerves is that subjects may deduct that this consists of a control/sham intervention if the upper limb performance is tested, or if the upper limb training is provided as part of the experimental protocol. Further studies are necessary to compare patients' perceptions about different types of controls for RPSS.

In summary, the results of the protocol “Comparison between the mechanisms underlying the effects of peripheral repetitive stimulation on upper limb motor performance in the subacute and chronic phases after stroke” will be relevant to inform future clinical trials in order to tailor RPSS to patients more likely to benefit from this intervention, in early or chronic stages. In addition, this study will provide preliminary data about mechanisms underlying effects of this intervention on GABA levels in the primary motor cortex, BOLD activation, and CBF perfusion in different stages after stroke.

## Ethics Statement

The studies involving human participants were reviewed and approved by Comitê de Ética em Pesquisa, Hospital Israelita Albert Einstein. The patients/participants provided their written informed consent to participate in this study.

## Author Contributions

AC, BH, EA, GS, and IS contributed to conception and design of the study. AC wrote the first draft of the manuscript. DW, FZ, GR, JK, MO, and RA wrote sections of the manuscript. All authors contributed to manuscript revision, read, and approved the submitted version.

## Funding

This work was funded by Fundação de Amparo à Pesquisa do Estado de São Paulo (FAPESP—grant 2018/03737-8). MO received a grant from FAPESP (2019/27859-8). FZ received a grant from MISTI (MIT International Science and Technology Initiatives) through MIT-Brazil Remote Internship—Experiential Learning Opportunities (ELO). AC received a grant from Conselho de Desenvolvimento Científico e Tecnológico (CNPQ, productivity grant 303070/2019-6). The funding source had no role in the design of this research and will not have any role during its execution, analyses, interpretation of the data, or decision to submit results.

## Conflict of Interest

DW was a shareholder of Translational MRI LLC, which provided the software for post-processing of pCASL data. The remaining authors declare that the research was conducted in the absence of any commercial or financial relationships that could be construed as a potential conflict of interest.

## Correction Note

A correction has been made to this article. Details can be found at: 10.3389/fneur.2025.1713834.

## Publisher's Note

All claims expressed in this article are solely those of the authors and do not necessarily represent those of their affiliated organizations, or those of the publisher, the editors and the reviewers. Any product that may be evaluated in this article, or claim that may be made by its manufacturer, is not guaranteed or endorsed by the publisher.

## Footnotes

^1^FMRIB's Software Library, https://www.fmrib.ox.ac.uk/fsl

^2^Glossary of Common Site Terms, https://clinicaltrials.gov/ct2/about-studies/glossary.
