# Correction: Effects of repetitive peripheral sensory stimulation in the subacute and chronic phases after stroke: study protocol for a pilot randomized trial

**DOI:** 10.3389/fneur.2025.1713834

**Published:** 2025-10-17

**Authors:** Jéssica Borges Kroth, Benjamim Handfas, Glaucia Rodrigues, Francisco Zepeda, Marco Aurélio Oliveira, Danny J. J. Wang, Raymundo Machado de Azevedo Neto, Gisele Sampaio Silva, Edson Amaro, Isaac Olubunmi Sorinola, Adriana Bastos Conforto

**Affiliations:** ^1^Hospital Israelita Albert Einstein, São Paulo, Brazil; ^2^Biological Engineering Department, Massachusetts Institute of Technology, Boston, MA, United States; ^3^Department of Neurology, Keck School of Medicine, University of Southern California, Los Angeles, CA, United States; ^4^Department of Physiotherapy, King's College London, London, United Kingdom

**Keywords:** sensory stimulation, stroke, rehabilitation, upper limb, nerve stimulation

In the published article, there was an error in Figure 1 as published. Three performance measures—Jebsen-Taylor test, pinch strength, and grasp strength—were obtained at three time points: before repetitive peripheral sensory stimulation, after stimulation, and after training. The original figure showed only two time points. The corrected Figure 1 and caption are provided below.

**Figure 1:**
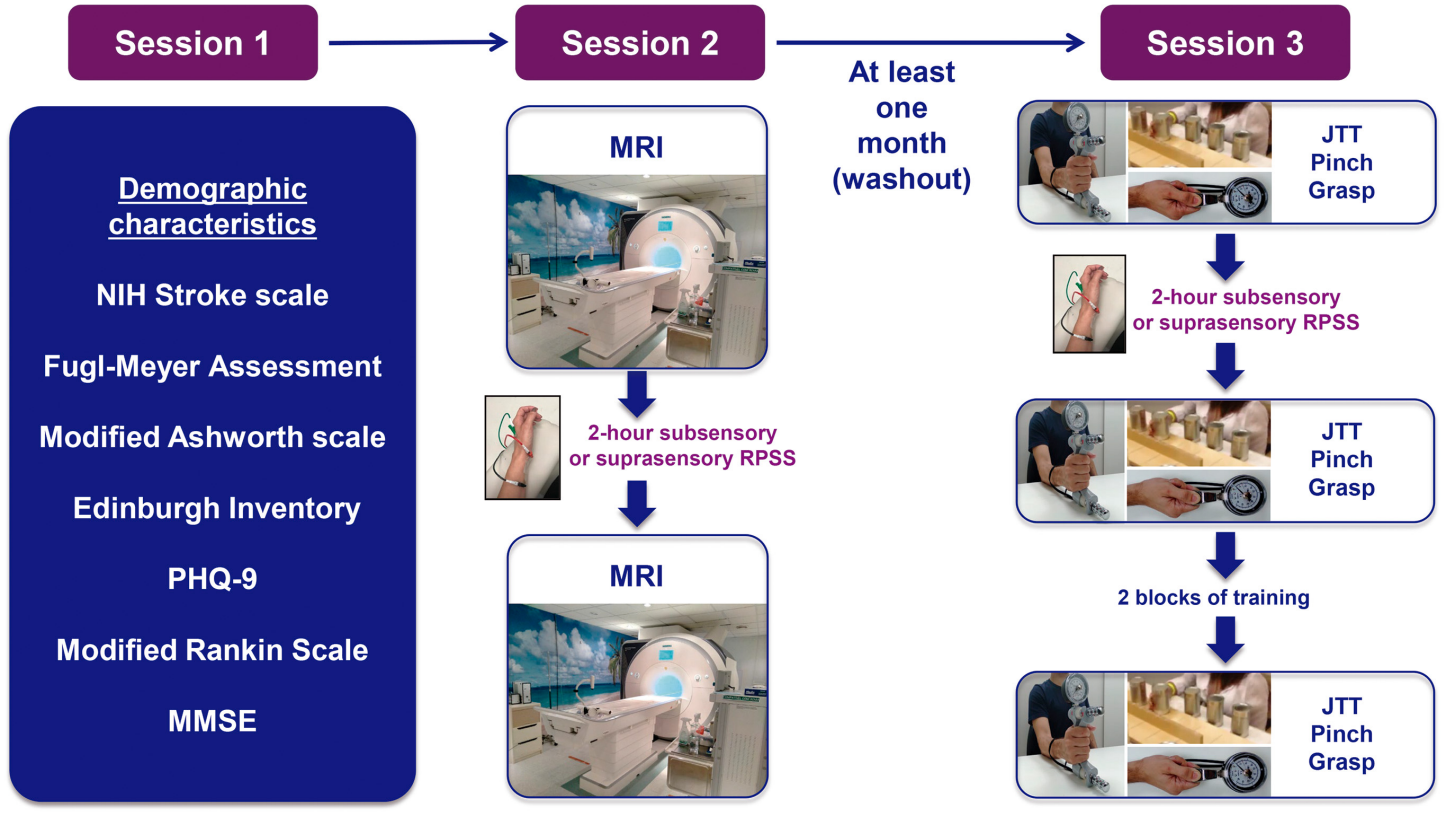
Experimental paradigm. NIH, national institutes of health; PHQ-9, patient health questionnaire-9; MMSE, mini-mental state exam; MRI, magnetic resonance imaging; RPSS, repetitive peripheral nerve sensory stimulation; JTT, Jebsen–Taylor test.

The original version of this article has been updated.

