# Supplementary material for: Effects of Repetitive Peripheral Sensory Stimulation in the Subacute and Chronic Phases After Stroke: Study Protocol for a Pilot Randomized Trial
Source: Front Neurol. 2022 Feb 16;13:779128. doi: 10.3389/fneur.2022.779128 (PMC8888931; doi:10.3389/fneur.2022.779128)
Supplement: Supplementary file 1 [file Data_Sheet_1.docx]

Supplementary Material

| Supplementary Figure 1. SPIRIT 2013 Checklist. | ...…..……………………………………………02 |
| --- | --- |
| **Supplementary Figure 2. SPIRIT flow diagram: Schedule of enrolment, interventions and assessments.** | ….………………………………………………07 |
| Supplementary Table 2. World Health Organization Trial Registration Data Set | …...….………………………………….………09 |
| Supplementary Data 1. Resonance imaging questionnaire | …...….………………………………….………12 |
| **Supplementary Data 2. Script for analysis - GABA spectroscopy data** | …...….………………………………………….14 |

Supplementary Figure 1. SPIRIT 2013 Checklist.

| **SPIRIT 2013 Checklist: Recommended Items to Address in a Clinical Trial Protocol and Related Documents** | | |
| --- | --- | --- |
| **Section/item** | **ItemNo** | **Description** |
| **Administrative information** | | |
| Title | 1 | Effects of repetitive peripheral sensory stimulation in the subacute and chronic phases after stroke: study protocol for a pilot randomized trial. (see page 1) |
| Trial registration | 2a | ClinicalTrials.gov (see page 2) |
|  | 2b | All items from the World Health Organization Trial Registration Data Set. (see Supplementary Material. Supplementary Table 2) |
| No Protocol version | 3 | Date and version identifier. (see page 5, lines 481-482) |
| Funding | 4 | Sources and types of financial, material, and other support. (see page 08, lines 890-900) |
| Roles and responsibilities | 5a | Names, affiliations, and roles of protocol contributor. (see page 1, lines 19-25) |
|  | 5b | Name and contact information for the trial sponsor. (see Supplemental Material. Supplementary Table 2) |
|  | 5c | Role of study sponsor and funders, if any, in study design; collection, management, analysis, and interpretation of data; writing of the report; and the decision to submit the report for publication, including whether they will have ultimate authority over any of these activities. (see page 08, lines 890-900) |
|  | 5d | Composition, roles, and responsibilities of the coordinating centre, steering committee, endpoint adjudication committee, data management team, and other individuals or groups overseeing the trial, if applicable (see Item 21a for data monitoring committee). (see page 3, lines 276-284) |
| **Introduction** |  |  |
| Background and rationale | 6a | Description of research question and justification for undertaking the trial, including summary of relevant studies (published and unpublished) examining benefits and harms for each intervention. (see pages 2-3, lines 124-259) |
|  | 6b | Explanation for choice of comparators. (see page 2, lines 160-190) |
| Objectives | 7 | Specific objectives or hypotheses. (see page 3, lines 246-259) |
| Trial design | 8 | Description of trial design including type of trial (eg, parallel group, crossover, factorial, single group), allocation ratio, and framework (eg, superiority, equivalence, noninferiority, exploratory). (see page 3, lines 264-267 and page 4-5, lines 452-457) |
| **Methods: Participants, interventions, and outcomes** | | |
| Study setting | 9 | Description of study settings (eg, community clinic, academic hospital) and list of countries where data will be collected. Reference to where list of study sites can be obtained. (see page 3, lines 270- 276) |
| Eligibility criteria | 10 | Inclusion and exclusion criteria for participants. If applicable, eligibility criteria for study centres and individuals who will perform the interventions (eg, surgeons, psychotherapists)**.** (see Table 1) |
| Interventions | 11a | Interventions for each group with sufficient detail to allow replication, including how and when they will be administered. (see pages 5, lines 476-543) |
|  | 11b | Criteria for discontinuing or modifying allocated interventions for a given trial participant (eg, drug dose change in response to harms, participant request, or improving/worsening disease). (see page 5 , lines 530-540) |
|  | 11c | - Strategies to improve adherence to intervention protocols, and any procedures for monitoring adherence (eg, drug tablet return, laboratory tests). Not applicable. |
|  | 11d | Relevant concomitant care and interventions that are permitted or prohibited during the trial. |
| Outcomes | 12 | Primary, secondary, and other outcomes, including the specific measurement variable (eg, systolic blood pressure), analysis metric (eg, change from baseline, final value, time to event), method of aggregation (eg, median, proportion), and time point for each outcome. Explanation of the clinical relevance of chosen efficacy and harm outcomes is strongly recommended. (see page 6, lines 546- 632) |
| Participant timeline | 13 | Time schedule of enrolment, interventions (including any run-ins and washouts), assessments, and visits for participants. A schematic diagram is highly recommended (see Figure). (see Supplementary Materials. Supplementary Figure 2) |
| Sample size | 14 | Estimated number of participants needed to achieve study objectives and how it was determined, including clinical and statistical assumptions supporting any sample size calculations. (see page 7, lines 690- 696 ) |
| Recruitment | 15 | Strategies for achieving adequate participant enrolment to reach target sample size. (see page 3, lines 305-312). |
| **Methods: Assignment of interventions (for controlled trials)** | | |
| Allocation: |  |  |
| Sequence generation | 16a | Method of generating the allocation sequence (eg, computer-generated random numbers), and list of any factors for stratification. To reduce predictability of a random sequence, details of any planned restriction (eg, blocking) should be provided in a separate document that is unavailable to those who enrol participants or assign interventions. (see page 4, lines 451-463) |
| Allocation concealment mechanism | 16b | Mechanism of implementing the allocation sequence (eg, central telephone; sequentially numbered, opaque, sealed envelopes), describing any steps to conceal the sequence until interventions are assigned. (see page 4, lines 451-463) |
| Implementation | 16c | Who will generate the allocation sequence, who will enrol participants, and who will assign participants to interventions. (see page 5, lines 464-472; 522-525) |
| Blinding (masking) | 17a | Who will be blinded after assignment to interventions (eg, trial participants, care providers, outcome assessors, data analysts), and how. (see page 7, lines 720-722) |
|  | 17b | If blinded, circumstances under which unblinding is permissible, and procedure for revealing a participant’s allocated intervention during the trial. (see page 5, lines 461-462) |
| **Methods: Data collection, management, and analysis** | | |
| Data collection methods | 18a | Plans for assessment and collection of outcome, baseline, and other trial data, including any related processes to promote data quality (eg, duplicate measurements, training of assessors) and a description of study instruments (eg, questionnaires, laboratory tests) along with their reliability and validity, if known. Reference to where data collection forms can be found, if not in the protocol. (see page 7, lines 698-722) |
|  | 18b | - Plans to promote participant retention and complete follow-up, including list of any outcome data to be collected for participants who discontinue or deviate from intervention protocols. Not applicable. |
| Data management | 19 | Plans for data entry, coding, security, and storage, including any related processes to promote data quality (eg, double data entry; range checks for data values). Reference to where details of data management procedures can be found, if not in the protocol. (see page, lines 698-722) |
| Statistical methods | 20a | Statistical methods for analysing primary and secondary outcomes. Reference to where other details of the statistical analysis plan can be found, if not in the protocol (see page 7-8, lines 724-768) |
|  | 20b | Methods for any additional analyses (eg, subgroup and adjusted analyses). (see page (see page 8, lines 756-766) |
|  | 20c | - Definition of analysis population relating to protocol non-adherence (eg, as randomised analysis), and any statistical methods to handle missing data (eg, multiple imputation). Not applicable. |
| **Methods: Monitoring** | | |
| Data monitoring | 21a | Composition of data monitoring committee (DMC); summary of its role and reporting structure; statement of whether it is independent from the sponsor and competing interests; and reference to where further details about its charter can be found, if not in the protocol. Alternatively, an explanation of why a DMC is not needed. (see page 8, lines 897-901) |
|  | 21b | - Description of any interim analyses and stopping guidelines, including who will have access to these interim results and make the final decision to terminate the trial. Not applicable. |
| Harms | 22 | Plans for collecting, assessing, reporting, and managing solicited and spontaneously reported adverse events and other unintended effects of trial interventions or trial conduct. (see page 5, lines 530-540 ) |
| Auditing | 23 | Frequency and procedures for auditing trial conduct, if any, and whether the process will be independent from investigators and the sponsor. (see page 7, lines 713-722) |
| **Ethics and dissemination** | | |
| Research ethics approval | 24 | Plans for seeking research ethics committee/institutional review board (REC/IRB) approval. (see page 8, lines 875-878) |
| Protocol amendments | 25 | Plans for communicating important protocol modifications (eg, changes to eligibility criteria, outcomes, analyses) to relevant parties (eg, investigators, REC/IRBs, trial participants, trial registries, journals, regulators) (see page and 5 lines 145-146 and page 3, lines 281-284) |
| Consent or assent | 26a | Who will obtain informed consent or assent from potential trial participants or authorised surrogates, and how (see Item 32). (see page 8, lines 874-878) |
|  | 26b | - Additional consent provisions for collection and use of participant data and biological specimens in ancillary studies, if applicable. Not applicable. |
| Confidentiality | 27 | How personal information about potential and enrolled participants will be collected, shared, and maintained in order to protect confidentiality before, during, and after the trial. (see page 7, lines 700-709) |
| Declaration of interests | 28 | Financial and other competing interests for principal investigators for the overall trial and each study site. (see page 11, lines 1141-1142) |
| Access to data | 29 | Statement of who will have access to the final trial dataset, and disclosure of contractual agreements that limit such access for investigators. (see page 7, lines 718-722) |
| Ancillary and post-trial care | 30 | Provisions, if any, for ancillary and post-trial care, and for compensation to those who suffer harm from trial participation. (see page 5, lines 538-540) |
| Dissemination policy | 31a | Plans for investigators and sponsor to communicate trial results to participants, healthcare professionals, the public, and other relevant groups (eg, via publication, reporting in results databases, or other data sharing arrangements), including any publication restrictions. |
|  | 31b | Authorship eligibility guidelines and any intended use of professional writers. |
|  | 31c | - Plans, if any, for granting public access to the full protocol, participant-level dataset, and statistical code. Not applicable. |
| **Appendices** |  |  |
| Informed consent materials | 32 | Model consent form and other related documentation given to participants and authorised surrogates. |
| Biological specimens | 33 | - Plans for collection, laboratory evaluation, and storage of biological specimens for genetic or molecular analysis in the current trial and for future use in ancillary studies, if applicable. Not applicable. |
| - Not applicable | | |

Supplementary Figure 2**.** SPIRIT flow diagram: Schedule of enrolment, interventions and assessments.

|  | **Enrolment** | **Allocation** | **Pre-intervention**  **RPSS** | **Intervention**  **RPSS** | **Post- intervention**  **RPSS** | **WASHOUT** | **Pre-intervention**  **RPSS** | **Intervention**  **RPSS** | **Post- intervention**  **RPSS** | **Intervention**  **Training** | **Post-intervention**  **Training** |
| --- | --- | --- | --- | --- | --- | --- | --- | --- | --- | --- | --- |
| **TIMEPOINT** | **^-T1^** | **^T0^** | **^T1^** | ***^T1^*** | ***^T1^*** | ***T1+ one month*** | ***^T2^*** | ***^T2^*** | ***^T2^*** | ***^T2^*** | ***^T2^*** |
| **ENROLMENT:** |  |  |  |  |  |  |  |  |  |  |  |
| **Eligibility screen** | X |  |  |  |  |  |  |  |  |  |  |
| **Demographic characteristics** | X |  |  |  |  |  |  |  |  |  |  |
| **Informed consent** | X |  |  |  |  |  |  |  |  |  |  |
| **Randomization** |  | X |  |  |  |  |  |  |  |  |  |
| **Modified Rankin Scale** |  | X |  |  |  |  |  |  |  |  |  |
| **NIHSS** |  | X |  |  |  |  |  |  |  |  |  |
| **MAS** |  | X |  |  |  |  |  |  |  |  |  |
| **Oldfield Inventory** |  | X |  |  |  |  |  |  |  |  |  |
| **FMA (upper limb)** |  | X |  |  |  |  |  |  |  |  |  |
| **MMSE** |  | X |  |  |  |  |  |  |  |  |  |
| **PHQ-9** |  | X |  |  |  |  |  |  |  |  |  |
| **EHI** |  | X |  |  |  |  |  |  |  |  |  |
| **INTERVENTIONS:** |  |  |  |  |  |  |  |  |  |  |  |
| ***Subsensory or suprasensory RPSS*** |  |  |  | X |  |  |  | X |  |  |  |
| ***Training*** |  |  |  |  |  |  |  |  |  | X |  |
| **ASSESSMENTS:** |  |  |  |  |  |  |  |  |  |  |  |
| ***JTT performance*** |  |  |  |  |  |  | X |  | X |  | X |
| ***Hand strength*** |  |  |  |  |  |  | X |  | X |  |  |
| ***GABA spectroscopy*** |  |  | X |  | X |  |  |  |  |  |  |
| ***BOLD fMRI*** |  |  | X |  | X |  |  |  |  |  |  |
| ***Arterial spin labeling*** |  |  | X |  | X |  |  |  |  |  |  |

*NIHSS: National Institutes of Health Stroke Scale; MAS: Modified Ashworth Scale; FMA: Fugl-Meyer Assessment of Sensorimotor recovery; MMSE: Mini-mental State Examination; PHQ-9: Patient Health Questionnaire-9; EHI: Edinburgh Handedness Inventory; RPSS: Repetitive peripheral nerve sensory stimulation; JTT: Jebsen-Taylor test; GABA: γ-Aminobutyric acid; BOLD fMRI: Blood-oxygenation level dependen functional magnetic resonance imaging. **Session two is only conducted at the São Paulo centers (Hospital Israelita Albert Einstein and Hospital das Clínicas da Faculdade de Medicina da Universidade de São Paulo)

Supplementary Table 2**.** World Health Organization Trial Registration Data Set

| **Trial registration data- World Health Organization Trial Registration Data Set** | |
| --- | --- |
| **Data Category** | **Information** |
|  | |
| Primary registry and trial identifying number | ClinicalTrials.gov NCT03956407 |
| Date of registration in primary registry | May 20, 2019 |
| Secondary identifying numbers | 3019629 |
| Source(s) of monetary or material support | Fundação de Amparo à Pesquisa do Estado de São Paulo, Hospital Israelita Albert Einstein |
| Primary sponsor | Hospital Israelita Albert Einstein |
| Secondary sponsor(s) | Fundação de Amparo à Pesquisa do Estado de São Paulo |
| Contact for public queries | Dr Adriana Conforto [adrianabc@einstein.br] |
| Contact for scientific queries | Dr Adriana Conforto [adrianabc@einstein.br] |
| Public title | Repetitive Nerve Stimulation to Improve Recovery After Stroke (RESTORES) |
| Scientific title | Comparison Between Mechanisms Underlying Effects of Repetitive Peripheral Nerve Stimulation on Upper Limb Motor Performance in the Subacute and Chronic Phases After Stroke |
| Countries of recruitment | Brazil |
| Health condition(s) or problem(s) studied | Stroke |
| Intervention(s) | Repetitive peripheral nerve stimulation |
| Key inclusion and exclusion criteria | Ages eligible for study: ≥18 years; Sexes eligible for study: both; Accepts healthy volunteers: no |
|  | Inclusion: Ischemic or hemorrhagic stroke confirmed by computed tomography or magnetic resonance imaging, between 7 days - 3 months before enrollment (subacute phase), and at least 6 months (chronic phase).  Ability to perform at least 4 of 7 tasks of the Jebsen-Taylor Test.  Upper limb paresis contralateral to the lesion. |
|  | Exclusion: Inability to provide informed consent, Anesthesia of the paretic hand, Severe spasticity at the paretic elbow, fist or fingers, defined by a score greater than 3 in the Modified Ashworth Scale; Shoulder pain or join deformity in the paretic limb, Lesions affecting the cerebellum or cerebellar pathways in the brainstem, Uncontrolled psychiatric disease, Neurological diseases such as Parkinson´s disease or chronic uncontrolled chronic disease such as cancer or cardiac failure, Aphasia or severe cognitive deficit |
| Study type | Interventional (Clinical Trial) |
|  | Allocation: Randomized |
|  | Primary purpose: Treatment |
| Date of first enrolment | December 1, 2019 |
| Target sample size | 72 participants |
| Recruitment status | Recruiting |
| Primary outcome(s) | Change in Jebsen-Taylor Test |
| Key secondary outcomes | Hand strength, cerebral blood flow assessed with arterial spin labeling, BOLD effect in the primary motor cortex (M1) assessed with functional magnetic imaging (fMRI) during a finger-tapping task performed with the paretic hand, and GABA levels in M1 evaluated with spectroscopy |

Supplementary Data 1. Resonance imaging questionnaire

**QUESTIONNAIRE PRIOR TO MAGNETIC RESONANCE IMAGING**

PATIENT IDENTIFIER N:

WEIGHT: _______ HEIGHT: _______

Read carefully all the information and answer the questions below:

These questions are made for a safe assessment of your Magnetic Resonance (MR) exam, please answer the questions as truthfully as possible. If there are any doubts, they can be clarified with the biomedical doctor or radiologist in the exam room.

1. Do you have or been subjected to:

Yes No- heart pacemaker or implantable cardioverter defibrillator

Yes No- aneurysm clip

Yes No- intracranial pressure monitor

Yes No- neurostimulator

Yes No- ocular prosthesis or any metal material inside the eye orbit

Yes No- orthopedic prosthesis or internal or external metallic fixation

Yes No- metal plate, stitches and sutures or any acupuncture material

Yes No- breast implant or expander

Yes No- hearing aid or metalic internal prosthesis

Yes No- metallic dental material (prosthesis, implants or braces)

Yes No- permanent tattoo or makeup in the last 15 days

Yes No- piercing, false eyelashes or metallic eyelash extension

Yes No- endoscopy or colonoscopy with clip placement in the last 7 days

Yes No- previous injury with metal shrapnel or firearm

Yes No- any other metallic or electronic device inside the body

Yes No- any kind of surgery

1. If you answered yes to any question above, please specify:

_________________________________________________________________________________________________________________________________________________________________________________________________________

1. Clinical safety:

Yes No- have you ever had an allergy to MRI contrast?

Yes No- do you have renal insufficiency?

Yes No- do you have severe asthma?

Yes No- previous allergy to medication or substances?

Yes No- medication was indicated before the exam? If so, did you take the indicated medication? Yes No

1. If you answered yes to any question above, please specify:

_________________________________________________________________________________________________________________________________________________________________________________________________________

1. Only for female patients:

Are you pregnant? Yes No

Are you brestfeeding? Yes No

If you are a woman of childbearing age, a pregnancy test will be made prior to the MRI.

Patients signature: ___________________________________

Supplementary Data 2. Script for analysis - GABA spectroscopy data

clear all

%% Script for autoprocessing MRS data based on folder architecture InCe

path_patient_folders = '/Volumes/Macintosh_HD3/Raymundo/RESTORES/1_Original_Data/1_Original_Data/';

patient_folders = dir([path_patient_folders 'P*']); % Retrieve names of patient folders

% NIFTI_FileNames = dir('**/*.nii'); % Retrieve names of NIFTI files

path_NIFTI = '/Volumes/Macintosh_HD3/Raymundo/RESTORES/2_Analysis/1_Subjects'; % path to NIFTI files

% NIFTI_count = 1;

for i = 1:length(patient_folders) % for every patient

disp(['Processing data from participant ', patient_folders(i).name])

current_patient = fullfile(path_patient_folders,patient_folders(i).name);

cd(current_patient)

%----------------PRE ANALYSIS-----------------%

cd('PRE')

cd('TWIX') % these two commands index into correct folder

NIFTI_FileNames = dir(fullfile(path_NIFTI,patient_folders(i).name,'PRE','uncompressed_nifti','*.nii')); % Get Nifti filename for this participant during PRE

NIFTI_FileName = NIFTI_FileNames(1).name;

%-----LEFT HEMISPHERE------%

MRS_struct = GannetLoad({dir('*LEFT*3mm_TOKYO.dat').name}, {dir('*LEFT*REF_TOKYO.dat').name});

MRS_struct = GannetFit(MRS_struct);

% MRS_struct = GannetCoRegister(MRS_struct, {fullfile(NIFTI_FileNames(NIFTI_count+1).folder, NIFTI_FileNames(NIFTI_count+1).name)});

MRS_struct = GannetCoRegister(MRS_struct, {fullfile(NIFTI_FileNames(1).folder, NIFTI_FileName)});

MRS_struct = GannetSegment(MRS_struct);

MRS_struct = GannetQuantify(MRS_struct);

GABA_results_PRE_LEFT(i) = MRS_struct.out.vox1.GABA.ConcIU_AlphaTissCorr_GrpNorm; % Extract GABA data into array

%-----RIGHT HEMISPHERE------%

MRS_struct = GannetLoad({dir('*RIGHT*3mm_TOKYO.dat').name}, {dir('*RIGHT*REF_TOKYO.dat').name});

MRS_struct = GannetFit(MRS_struct);

% MRS_struct = GannetCoRegister(MRS_struct, {fullfile(NIFTI_FileNames(NIFTI_count+1).folder, NIFTI_FileNames(NIFTI_count+1).name)});

MRS_struct = GannetCoRegister(MRS_struct, {fullfile(NIFTI_FileNames(1).folder, NIFTI_FileName)});

MRS_struct = GannetSegment(MRS_struct);

MRS_struct = GannetQuantify(MRS_struct);

GABA_results_PRE_RIGHT(i) = MRS_struct.out.vox1.GABA.ConcIU_AlphaTissCorr_GrpNorm;

%----------------POS ANALYSIS-----------------%

cd('..')

cd('..')

cd('POS')

cd('TWIX') % switch folder to

NIFTI_FileNames = dir(fullfile(path_NIFTI,patient_folders(i).name,'POS','uncompressed_nifti','*.nii')); % Get Nifti filename for this participant during PRE

NIFTI_FileName = NIFTI_FileNames(1).name;

%-----LEFT HEMISPHERE------%

MRS_struct = GannetLoad({dir('*LEFT*3mm_TOKYO.dat').name}, {dir('*LEFT*REF_TOKYO.dat').name});

MRS_struct = GannetFit(MRS_struct);

% MRS_struct = GannetCoRegister(MRS_struct, {fullfile(NIFTI_FileNames(NIFTI_count).folder, NIFTI_FileNames(NIFTI_count).name)});

MRS_struct = GannetCoRegister(MRS_struct, {fullfile(NIFTI_FileNames(1).folder, NIFTI_FileName)});

MRS_struct = GannetSegment(MRS_struct);

MRS_struct = GannetQuantify(MRS_struct);

GABA_results_POS_LEFT(i) = MRS_struct.out.vox1.GABA.ConcIU_AlphaTissCorr_GrpNorm;

%-----RIGHT HEMISPHERE------%

MRS_struct = GannetLoad({dir('*RIGHT*3mm_TOKYO.dat').name}, {dir('*RIGHT*REF_TOKYO.dat').name});

MRS_struct = GannetFit(MRS_struct);

% MRS_struct = GannetCoRegister(MRS_struct, {fullfile(NIFTI_FileNames(NIFTI_count).folder, NIFTI_FileNames(NIFTI_count).name)});

MRS_struct = GannetCoRegister(MRS_struct, {fullfile(NIFTI_FileNames(1).folder, NIFTI_FileName)});

MRS_struct = GannetSegment(MRS_struct);

MRS_struct = GannetQuantify(MRS_struct);

GABA_results_POS_RIGHT(i) = MRS_struct.out.vox1.GABA.ConcIU_AlphaTissCorr_GrpNorm;

cd('..')

cd('..')

cd('..')

% NIFTI_count = NIFTI_count + 2; % increase counter by 2, to access next 2 NIFTI files

clear MRS_struct

close all

end

%----------------EXPORT DATA TO SPREADSHEET-----------------%

output_table = table({patient_folders.name}', GABA_results_PRE_LEFT', ...

GABA_results_PRE_RIGHT', GABA_results_POS_LEFT', GABA_results_POS_RIGHT');

output_table.Properties.VariableNames = {'Patient' 'PRE LEFT' 'PRE RIGHT' 'POS LEFT' 'POS RIGHT'};

filename = '/Volumes/Macintosh_HD3/Raymundo/RESTORES/2_Analysis/2_Groups/Spectroscopy/results.xlsx';

writetable(output_table,filename,'Sheet'
